# Effect of the Essential Oil of *Minthostachys verticillata* (Griseb.) Epling and Limonene on Biofilm Production in Pathogens Causing Bovine Mastitis

**DOI:** 10.3389/fvets.2018.00146

**Published:** 2018-07-03

**Authors:** María F. Cerioli, Melina V. Moliva, Laura N. Cariddi, Elina B. Reinoso

**Affiliations:** ^1^Departamento de Microbiología e Inmunología, Facultad de Ciencias Exactas, Físico-Químicas y Naturales, Universidad Nacional de Rio Cuarto, Río Cuarto, Argentina; ^2^INBIAS CONICET, Río Cuarto, Argentina

**Keywords:** essential oil, limonene, *Minthostachys verticillata*, inhibitory effect, bovine mastitis

## Abstract

Bovine mastitis causes large annual economic losses around the world. Different microorganisms are associated with the disease. The capacity of pathogens to adhere to bovine mammary epithelial cells is associated with biofilm production which leads to antibiotic resistance. Research is now leading to search alternative control methods and medicinal plants constitute a natural, safe, effective and inexpensive option. *Minthostachys verticillata* is an autochthonous medicinal plant of Argentina with multiple ethnobotanical properties. In a previous study, we demonstrated that the essential oil (EO) of this species and limonene, one of its compounds, inhibited the growth of mastitis pathogens. The objective of the present work was to determine the inhibitory effect of the essential oil of *M. verticillata* and limonene, on biofilm formation and on mature biofilm produced by pathogens isolated from bovine mastitis. Time kill assay and bacterial lysis were also determined. Furthermore, RAPD-PCR assays were performed to determine changes in bacterial DNA after EO and limonene exposition. Bacterial isolates were identified as *Escherichia coli* (EC3 and EC9), *Bacillus pumilus* (BP5, BP6, and BP7) and *Enterococcus faecium* (EF1) by rRNA 16S sequencing and MALDI-TOF MS. All the strains were able to form biofilm. Addition of both lactose and sucrose did not affect biofilm production. MIC values for EO were 3.6 mg/ml for *E. faecium*; 0.9 mg/ml for *E. coli* (EC3), 14.5 mg/ml for *E. coli* (EC9), 1.8 mg/ml for *B. pumilus* (BP7), 3.63 mg/ml for *B. pumilus* (BP6) and 29.0 mg/ml for *B. pumilus* (BP7). MIC values for limonene were 6.6 mg/ml for *B. pumilus* (BP6) and 105 mg/ml for *B. pumilus* (BP5). These results demonstrated that EO was more effective than limonene, showing also bactericidal action against *E. faecium* (minimal inhibitory concentration (MBC) = 29.0 mg/ml). This result was corroborated by time of death assay, observing a cell decrease after at 6 h, and then by bacterial lysis assay. Both EO and limonene affected mature biofilm of isolated strains. The results contribute to the study of EO and limonene which may serve as a therapy against bovine mastitis pathogens inhibiting the development of pathogenic bacteria.

## Introduction

Bovine mastitis is a disease that causes large annual economic losses around the world. Different pathogens, classified as environmental and contagious, are associated with the disease as their ability to form biofilm leads to the advance of the infection (1). Reports showed that mastitis pathogens are able to form biofilms (2–4). The establishment of the biofilm depends on the ability of the pathogen to adhere to bovine mammary epithelial cells. Biofilm is composed of a matrix that protects the cells from unfavorable conditions. Mastitis agents growing on biofilm are more resistant to antimicrobial agents (5).

Whereas antibiotic therapy has a positive impact on the udder health and milk production, it is not always effective and leaves residues with implications for human health. In addition, the ability of pathogens to live in the mammary gland forming biofilm, would be a potential source of persistent or chronic infection (6). The ability to invade mammary epithelial cells and the intracellular survival of bacterial agents also has a role in the pathogenesis of persistent mastitis. Generally, these infections are difficult to treat (7). New approaches aim to generate and to increase immune protection in the sick udder during immunosuppressive periods, leading to a significant effect on resistance to infection (8).

Alternative control methods are currently being sought, and medicinal plants can be a natural, safe, effective and inexpensive option for the treatment of this disease. *Minthostachys verticillata* is an aromatic and autochthonous medicinal plant, native of Cordoba province, Argentina. It is one of the most used in folk medicine due to multiple ethnobotanical therapeutic properties (9). In a previous study, we demonstrated that the essential oil (EO) of this species and limonene, one of its compounds, inhibited the growth of *Streptococcus uberis* causing bovine mastitis (10). Additionally, we showed that EO and limonene also have antimicrobial effect on major mastitis pathogens such as *Staphylococcus aureus, Streptococcus uberis, Escherichia coli* and Coagulase-Negative Staphylococci (CNS) by disk diffusion assay (11).

The objective of the present work was to determine the inhibitory effect of the essential oil of *Minthostachys verticillata* and limonene, on biofilm formation and on mature biofilm produced by pathogens isolated from bovine mastitis. For this, minimal inhibitory concentration and minimal bactericide concentration (MCB) were evaluated. Time kill assay and bacterial lysis were also determined. Furthermore, Random Amplified of Polymorphic DNA (RAPD-PCR) assay was performed to determine changes in bacterial DNA after EO and limonene exposition.

## Materials and methods

### Essential oil extraction

The EO (δ = 0.929 g/ml) was obtained from the aerial parts of the plant by hydrodistillation according to Montironi et al. (10). The identification of the main components was carried out by Gas Chromatography-Mass Spectrometry (GC-MS) comparing the retention times of these compounds with those of standard drugs: pulegone, mentone, limonene, cineole, α-pinene, and β-pinene. GC-MS was performed by the service of Instituto Multidisciplinario de Biología Vegetal (IMIV-Conicet), Cátedra de Química Orgánica, Facultad de Ciencias Exactas, Físicas y Naturales, Universidad Nacional de Córdoba. Limonene was purchased from Sigma Aldrich (St. Louis, USA) as (*R*)-(+)-Limonene (δ = 0.840 g/ml).

To perform the different tests, EO and limonene first dissolved in DMSO 1:2 (1 part of EO or limonene and 2 parts of DMSO) and then in PBS 1:10 (1 part of mixture of EO or limonene with DMSO and 10 parts of PBS) were used. Serial dilutions (1/2) were performed resulting in concentrations from 929 to 0.45 mg/ml and 840 to 0.41 mg/ml for EO and limonene, respectively.

### Bacterial isolates

Fifteen milk samples were collected from cows, with subclinical mastitis, belonging to a dairy herd located in the central dairy region of Argentina 130 km away from Rio Cuarto city. The dairy herd was visited once during August 2016. The average herd size was 130 milking cows.

Samples were immediately refrigerated at 4°C and subjected to bacteriological analysis within 24 h of isolation. The isolates were cultured on blood agar plates with 5% bovine blood. Nine of them showed to have more than three different colony types, for which they were considered as contaminated according to Hogan et al. (12). Six samples, which had only one type of colony, were used in the present study. Then, isolates were cultured on Trypticase Soy Agar (TSA) (Britania) for 24 h at 37°C and were presumptively identified based on colonial appearance, Gram stain reaction and catalase test. Isolates were maintained frozen at −20°C in Trypticase Soy Broth (TSB) (Britania) containing 20% glycerol.

### Bacterial identification

Bacterial isolates were identified by conventional bacteriological methods and then confirmed by 16 rRNA -sequencing and additionally by matrix assisted laser desorption/ionization—time of flight mass spectrometry (MALDI-TOF MS system—Bruker Daltonik MALDI Biotyper). 16 rRNA -sequencing was carried out by CERELA-CONICET sequencing service. DNA sequence information was searched with Basic Local Alignment Search Tool (BLAST) using the NCBI database. MALDI-TOF analysis was performed by Microbiology Laboratory of the Hospital Privado Universitario de Córdoba service. In brief, a score of <1.700 was interpreted as no identification, 1.700–1.999 indicated an identification to genus level, 2.000–2.299 indicated a reliable identification of the genus and a probable species identification, and 2.300–3.000 represented a high probability of species identification.

### Minimal inhibitory concentration and minimal bactericide concentration assay

The microdilution method was employed to determine the Minimal Inhibitory Concentration (MIC) and MCB for EO and limonene against bacterial isolates according to the recommendations of the National Committee for Clinical Laboratory Standards as described by Montironi et al. (10).

Each concentration of EO or limonene was assayed four times and the experiment was repeated on 3 different occasions.

### Time kill assay

Decrease of bacteria was determined over several hours according to El Kolli et al. (13). A concentration corresponding to the MIC of the EO and limonene at a concentration that showed the best inhibitory effect were used.

### Bacterial lysis

The bacteriolytic action was measured by absorbance at OD_620_ nm according to El Kolli et al. (13). The concentration corresponding to the MIC, and limonene at a concentration that showed the best inhibitory effect were assayed.

### Random amplified polymorphic DNA (RAPD) analysis

RAPD-PCR assay was performed in order to detect DNA alterations after addition of EO and limonene MIC. Results were compared with RAPD-PCR assay without addition of EO and limonene. Approximately 25 ng of chromosomal DNA was used according to the optimized protocol in our lab (14). Primer P13- 5′-ACCGCCTGCT-3′ was used. A positive and a negative control were used in each run. Each isolate was assayed twice. Amplified products were determined by electrophoresis in 1.5% of agarose gel (Promega) in 0.5 X TBE at 90 V for 50 min. Gels stained with GelGreenTM were observed under UV light with MiniBisPRO gel documentation (BioAmerica, USA) and recorded. A 100 bp DNA marker (Promega) was used as a DNA molecular size standard. Band profiles were compared for similarity by visual inspection.

### Biofilm assays

The ability of the bacterial isolates to form biofilm *in vitro* on an abiotic surface was determined using a sterile 96-well flat bottom polystyrene plate as previously described (4). A *Staphylococcus epidermidis* strain was used as positive control and TSB as negative control. The effect of adding sugars as sucrose (5%) and lactose (5%) and milk compounds as skim milk powder (5%) (Oxoid^TM^) and casein hydrolysate (3 mg/ml) was determined. All the additives and casein hydrolysate were purchased at Sigma, St. Louis, MO, USA.

### Inhibitory effect of EO and limonene on biofilm formation

The activity of EO and limonene was evaluated in the prevention of biofilm formation. MIC levels of EO and limonene previously determined were added to a bacterial suspension of 10^6^ cfu/ml and the 96-well flat bottom polystyrene plates were incubated at 37° C for 24 h. Four wells in each plate containing only EO or limonene were used as negative controls. The wells were washed and colored according to Moliva et al. (4). The effect of MIC on biofilm formation was determined according to Aiemsaard et al. (15). Percentages of inhibition for each sample were determined by comparing the mean optical density of control wells (without EO or limonene added) with the bacterial suspension (with the addition of EO or limonene) using the following formula:

$$\begin{array}{l} \left[1-\left(\text{O}{\text{D}}_{560}\text{sample}/\text{O}{\text{D}}_{560}\text{control}\right)\right]\times 100\% \end{array}$$

Each concentration of EO or limonene was assayed four times and the experiment was repeated on 3 different occasions.

### Effect of EO and limonene on mature biofilm

The stability of the biofilm against EO and limonene in mature biofilms was determined according to the protocol described above, except that EO and limonene were added to the wells 24 h after biofilm formation. Absorbance at OD_560_ nm was read and percentages of inhibition were calculated.

### Statistical analysis

Statistical analysis was performed using Graphpad Prism. Analysis of variance (ANOVA) and the Tukey multiple comparison test were used. The nominal *p*-value for statistical significance was *p* ≤ 0.05.

## Results

### Essential oil extraction

Chromatographic profile of EO showed the presence of pulegone and menthone, identified as major components with percentages of 74.96 and 20.38%, respectively. Other compounds included were limonene (1.50%), α-pinene (0.48%) and β-pinene (0.50%). The sum of the relative percentages of the compounds identified in EO was 97.86%. The remaining percentage (2.14%) corresponds to compounds that could not be identified.

### Bacterial identification

The bacterial isolates were identified by conventional bacteriological methods and later confirmed by 16 rRNA sequencing and MALDI-TOF analysis. The isolates were identified as *Escherichia coli* (named EC3 and EC9), *Bacillus pumilus* (named BP5, BP6 and BP7) and *Enterococcus faecium* (named EF1).

### Minimal inhibitory concentration and minimal bactericide concentration

The antibacterial activity of EO and one of its component limonene was assayed against the bovine mastitis isolated. MIC and MBC of EO and limonene were determined. Results obtained showed that EO inhibited the growth of all isolates. On the other hand, limonene had an inhibitory effect on BP5 and BP6 isolates. MIC values for EO were 3.6 mg/ml for *E. faecium*; 0.9 mg/ml for *E. coli* (EC3), 14.5 mg/ml for *E. coli* (EC9), 1.8 mg/ml for *B. pumilus* (BP7), 3.63 mg/ml for *B. pumilus* (BP6) and 29.0 mg/ml for *B. pumilus* (BP7). MIC values for limonene were 6.6 mg/ml for *B. pumilus* (BP6) and 105 mg/ml for *B. pumilus* (BP5).

On the other hand, EO had bactericidal effect on EF1 isolate at a high concentration (MBC = 29.0 mg / ml) and limonene had no bactericidal action on any isolate (Table 1).

**Table 1:** Minimal inhibitory concentration (MIC) of EO and limonene.

	**Minimum inhibitory concentration (MIC)**	
	**EO (mg/ml)**	**Limonene (mg/ml)**
*Enterococcus faecium* (EF1)	3.63	–
*Escherichia coli* (EC3)	14.51	–
*Escherichia coli* (EC9)	0.90	–
*Bacillus pumilus* (BP5)	29.0	105
*Bacillus pumilus* (BP6)	3.63	6.6
*Bacillus pumilus* (BP7)	1.8	–

*(–) = no MIC was observed*.

### Time kill assay

The time of death was evaluated by bacterial decrease in all the isolates treated with the MIC of EO and limonene or the concentration of limonene that showed the best inhibitory effect against bacteria.

**Figure 1:**
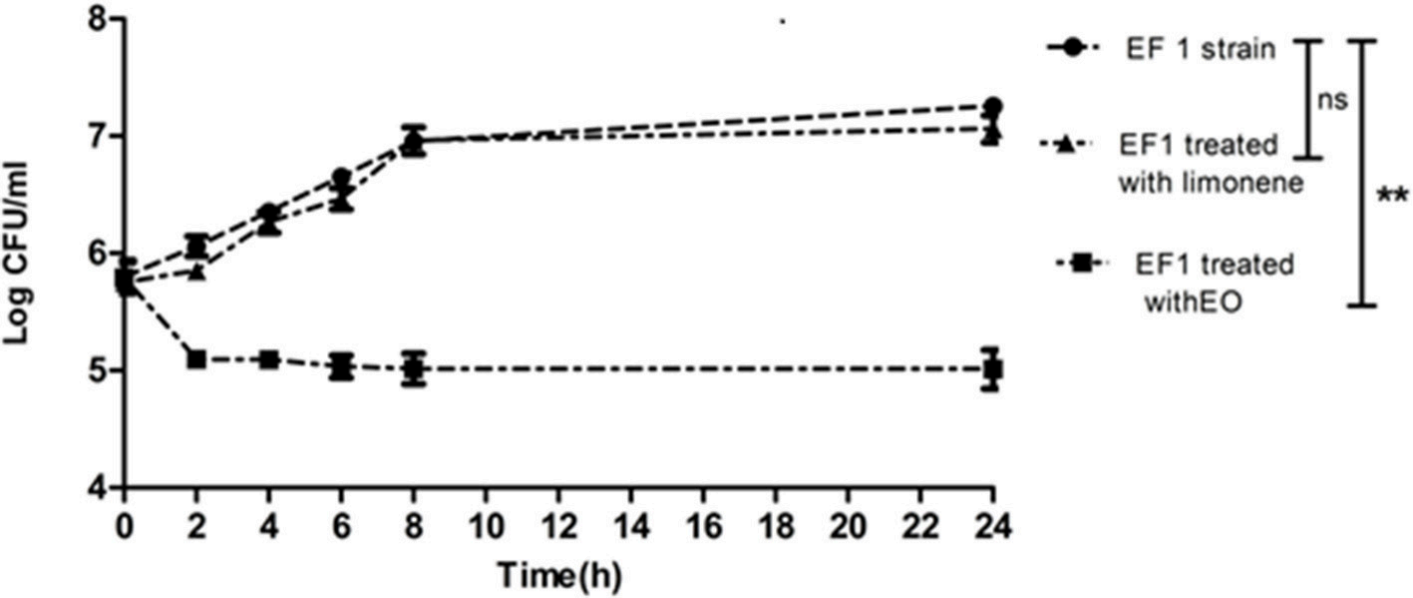
Bacterial counts of EF1 isolate after MIC of EO and limonene at concentration that showed the best inhibitory effect against EF1. ^**^p < 0.01 compared with EF1 alone. Each value represents means ± SD.

Results showed that EF1 strain treated with the MIC of EO, significantly reduced the population after 6 h of treatment (*p* < 0.01). On the other hand, limonene showed no effect (Figure 1).

The effect of EO and limonene was also evaluated in isolates where these compounds did not show bactericidal action. No difference was observed in bacterial count at the different times assayed.

### Bacterial lysis

**Figure 2:**
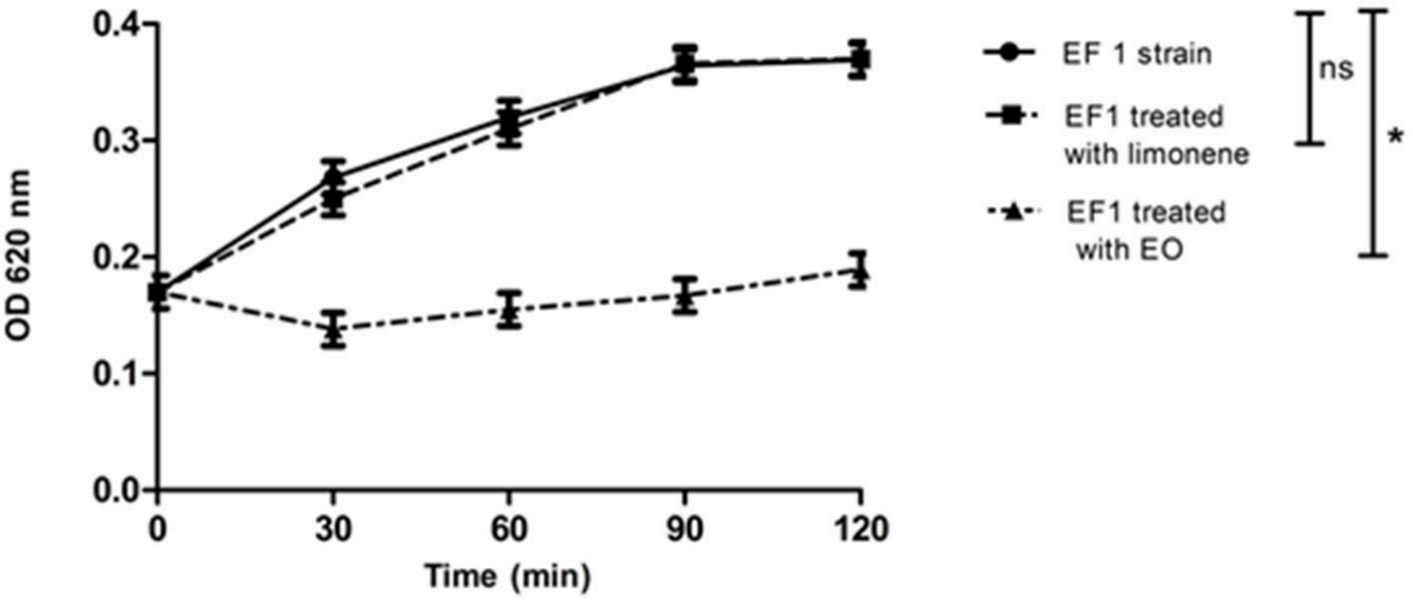
Effect of EO and limonene on bacterial lysis. ^*^p < 0.05 compared with EF1 alone. Each value represents means ± SD.

Bacterial lysis was determined in EF1 isolate, evaluating the addition effect of the MIC of EO or the limonene concentration that showed the best inhibitory effect against EF1 (52.5 mg/ml). Results obtained showed that EO was able to produce 50% of bacterial lysis (*p* < 0.05) after 30 min (Figure 2). On the other hand, limonene did not produce bacterial lysis.

Furthermore, EO and limonene were also tested for lysis production on the remaining isolates and no bacterial lysis was observed. In addition, the activity of the EO against the bacteria tested was assayed over the time without achieving at the absence of viable forms, except for EF1.

### Random amplified polymorphic DNA (RAPD) analysis

**Figure 3:**
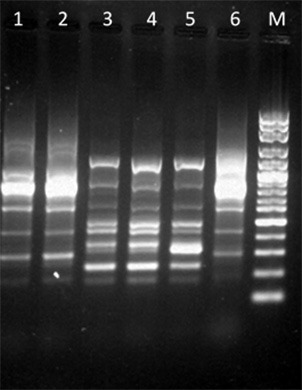
RAPD-PCR profiles among isolates after addition of EO or limonene. Lane 1: EC9 after addition of EO; lane 2: EC9 after addition of limonene; lane 3: EF1 after addition of EO; lane 4: EF1 after addition of limonene; lane 5: EF1 isolate without treatment; lane 6: EC9 isolate without treatment; lane M: Molecular marker, 100 bp DNA ladder.

Variations in RAPD profiles after addition of MIC of EO and limonene at the concentration that showed the best inhibitory effect was observed as variations in band intensity in the isolates assayed. Figure 3 shows the RAPD-PCR profiles of the EC9 and EF1 isolates after adding EO or limonene.

### Biofilm assays

**Figure 4:**
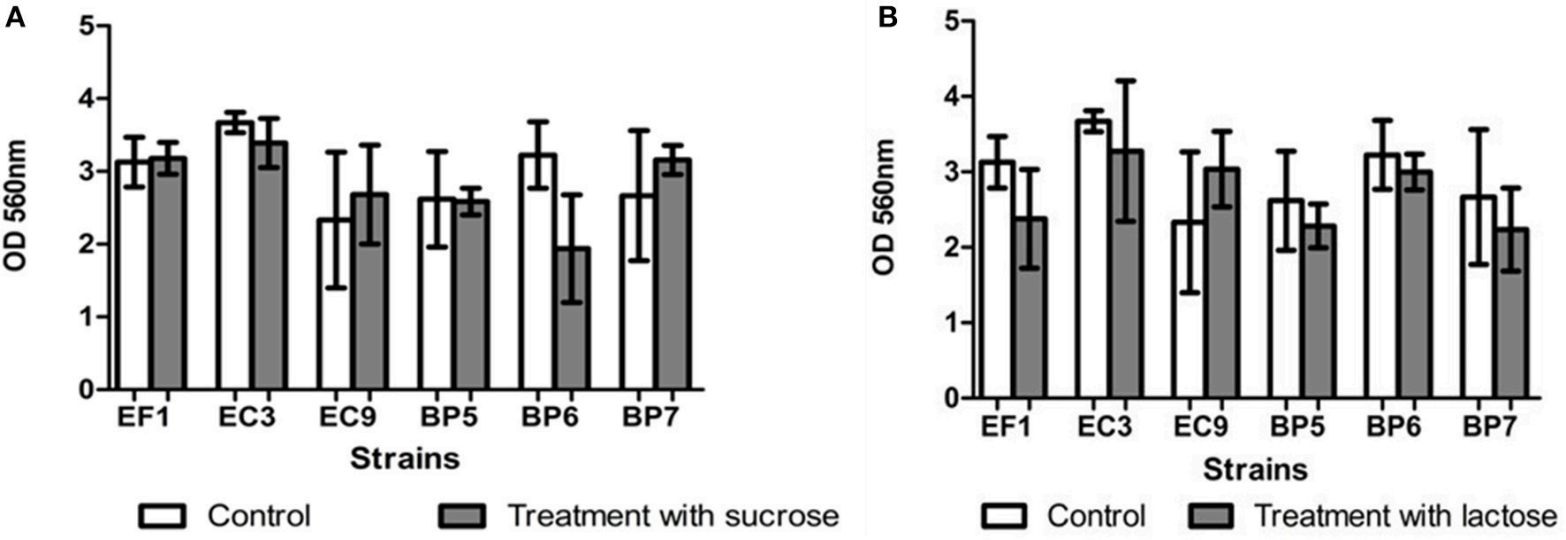
Effect of addition of sucrose **(A)** and lactose **(B)** on biofilm formation. Each value represents means ± SD.

Results showed that all the isolates were able to form biofilm *in vitro*. Addition of lactose or sucrose to the culture media did not affect biofilm production. *B. pumilus* (BP6) showed a decrease in the biofilm production, although it seems to affect biofilm production, it is not statistically significant respect to the control. Figure 4 shows the effect of each carbohydrate addition.

**Figure 5:**
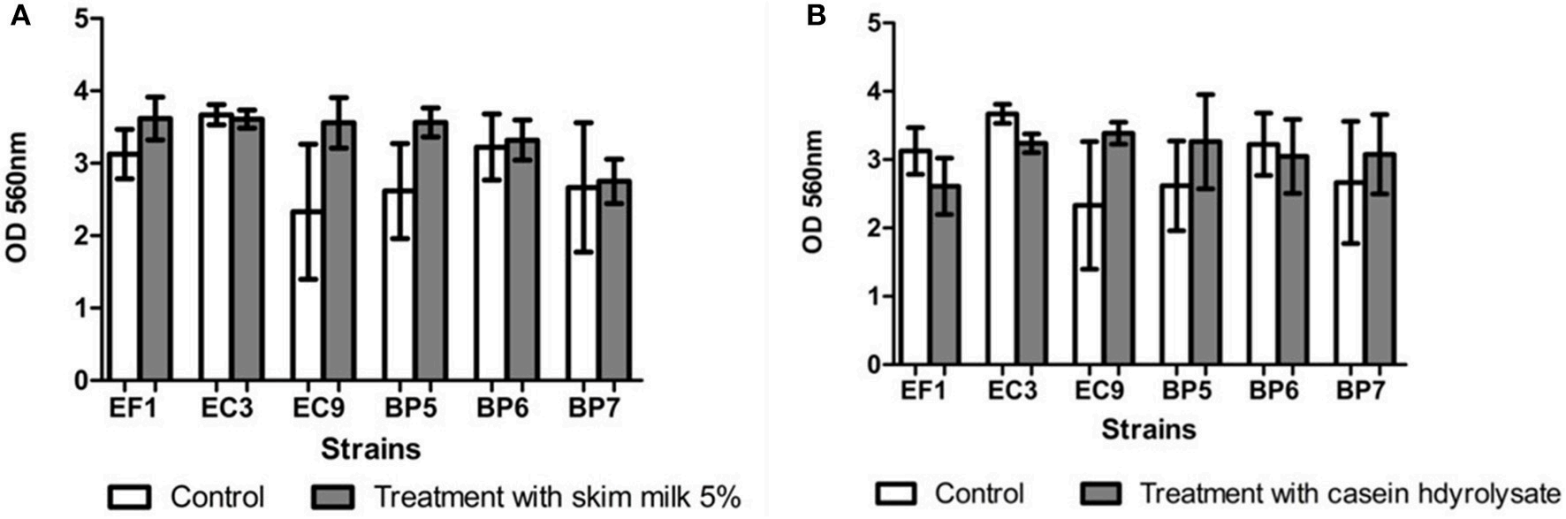
Effect of addition of skim milk **(A)** and lactose casein hydrolysate **(B)**. Each value represents means ± SD.

Addition of skim milk showed a slight increase in biofilm production in isolates EF1, BP5, BP7, and EC9, but no statistical difference was found. Similar results were obtained after addition of casein hydrolysate in strains BP5, BP7, and EC9 which showed a minor increase in biofilm production (Figure 5).

### Effect of EO and limonene during biofilm formation and on mature biofilm

The action of EO and limonene during biofilm formation and on mature biofilm was studied using MICs previously determined. In the isolates in which it was not possible to determine the MIC of limonene, the concentration that showed the best inhibitory effect against bacteria was used (52.5 mg/ml for EF1, 210 mg/ml for EC3, 105 mg/ml for EC9 and 210 mg/ml for BP7).

**Figure 6:**
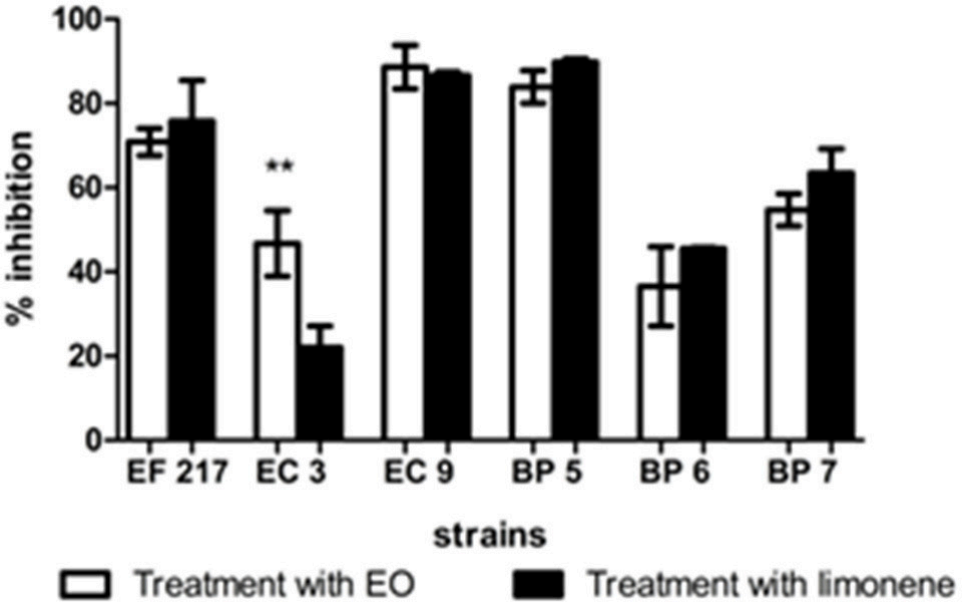
Inhibitory effects of EO and limonene during biofilm formation. ^**^p < 0.01 compared with limonene. Each value represents means ± SD.

Inhibition percentages among 36.51 and 89.60% were observed after EO addition, between 22.06 and 89.83% of inhibition was observed when limonene was added during biofilm formation (Figure 6).

**Figure 7:**
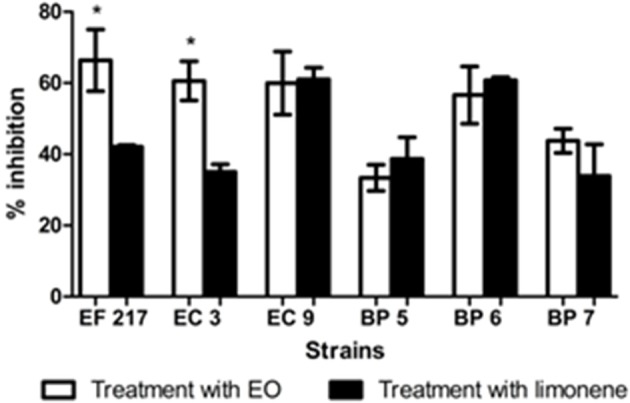
Inhibitory effects of EO and limonene on mature biofilm ^*^p < 0.05 compared with limonene. Each value represents means ± SD.

Inhibition percentages of EO from 35.06 to 66.35%, and limonene from 33.36 to 61% were observed on mature biofilm were observed on mature biofilm (Figure 7).

## Discussion

The drawbacks of antibiotic therapy and the difficulties in eliminating infections associated with the biofilm have guided the search for alternative therapeutic agents. Previously, we have demonstrated the antibacterial activity of both EO and limonene on pathogens isolated from bovine mastitis as *Staphylococcus aureus* and coagulase negative staphylococci (11). In the present work, the inhibitory effect of the *M. verticillata* EO and limonene was determined on mastitis pathogens able to form biofilm *in vitro*.

The chromatographic profile of EO was similar to a previously one reported by Montironi et al. (10), with a predominantly pulegone-menthone chemotype.

All bacterial isolates identified in the present work correspond to environmental pathogens. Methods to control these bacteria sometimes are inadequate (16). *Enterococcus* spp. are part of the normal flora of urogenital and gastrointestinal systems of mammals. They live in different niches as soil, natural waters and plants. *E. faecium* and *Enterococcus faecalis* are cause of bovine mastitis (17). Enterococcus group is important due to their ability to transfer conjugative plasmids (18) increasing the virulence by antibiotic resistance and leading to an important problem in the therapeutic treatment.

*E. coli* is found in organic materials as dung and bedding. The contact with organic matter and the milking process favor the infection of the udder. The proportion of E. coli as a causative agent in bovine clinical mastitis varies between countries. Bovine mastitis caused by *E. coli* can range from mild to moderate clinical signs and usually have a natural cure (19, 20). Although, sometimes can produced a severe systemic disease.

However, *E. coli* strains may turn into chronic or recurrent infections and antimicrobial therapy with the use of fluoroquinolones and cephalosporins becomes necessary. Nevertheless, both antibiotics should be used in animals destined for food under specific indications and after a microbiological diagnosis (21).

*Bacillus* spp. comprise a varied group of bacteria disseminated in soil and water environment. They could infect the mammary gland when cows have access to pasture or through dirty infusions prior to intramammary treatment or dry cow therapy. Reports have showed that different *Bacillus* species including *B. pumilus* are able to cause infections (22). Nieminen et al. (23) could identify *Bacillus* as the major organism in 23 mastitic milk samples where four of them were *B. pumilus*. Similarly, Amer et al. (24) found *Bacillus* spp. in 22.1% of isolates. The presence of *Bacillus* spp. may risk the quality guarantee of milk products.

The results of MIC and MBC values showed that EO has antibacterial activity against clinical isolate bovine mastitis pathogens. MIC results showed that EO was able to inhibit the growth of all isolates tested in lower concentrations than limonene, demonstrating that EO was the most effective. Furthermore, in this study it was observed that concentrations of EO required to inhibit most of the Gram-positive bacteria were lower than those required to inhibit the growth of Gram-negative bacteria, except for BP5 and EC9. On the other hand, limonene only had inhibitory action on Gram-positive bacteria tested. The results obtained were similar to those reported by other authors. Klancnik et al. (25) determined MIC and MBC values of four extracts of rosemary (*Rosmarinus officinalis*) soluble in oil and water, against Gram positive (*Bacillus* spp. and *Staphylococcus* spp.) and Gram negative bacteria (*Campylobacter* spp. and *Salmonella* spp.) showing that Gram-positive bacteria were more sensitive than Gram-negative bacteria.

Results of time of death assays are according to those observed in the MBC test, in which the bactericidal action of EO was observed on EF1 isolate. On the other hand, it was found that limonene, which had not shown bactericidal action in EF1 isolate, also failed significantly to decrease the bacterial population.

No difference was observed in bacterial count at different times assayed after the addition of EO and limonene in isolates where these compounds did not show bactericidal action.

Results of lysis production are in agreement with those of El Kolli et al. (13) who reported that the absorbance relative values decreased significantly to 35.3% for *Bacillus cereus* and 40% for *Proteus mirabilis* when the essential oil of *Daucus gracilis* was studied.

Variations in RAPD profiles were reported by Hamedo (26) who studied the antimicrobial activity of the essential oils of *Rosmarinus officinalis*. The authors found that differences in RAPD profiles could be due to DNA damage after essential oils addition, although the differences could also be attributed to the oligonucleotide used (26). Changes in bands intensity were found in this study, suggesting no DNA damage.

In the present study, all the isolates were able to form biofilm, although the addition of carbohydrates did not affect biofilm production. Similar results were obtained by Moliva et al. (4), who reported that addition of lactose (0.5 and 5%) in the culture medium did not promote biofilm formation in *S. uberis* isolates. However, results are in contrast to those reported by Abureema (3) who informed that that addition of lactose decreased biofilm formation and the addition of fructose, glucose, or sucrose increased biofilm formation by *S. uberis*.

Results obtained after addition of skim milk or α-casein are in accordance with those reported by Tasi et al. (27), who demonstrated that *S. uberis* isolates grown in BME-UV1 complete medium with the addition of casein did not increase biofilm production. Likewise, Atulya et al. (28) showed milk components did not affect biofilm formation in *E. coli* and *S. aureus* isolates. According to the literature, several factors and additives can influence the formation of biofilm in the different genera of bacteria.

The effects of EO in inhibiting biofilm formation was determined. Addition of both EO and limonene significantly reduced the production of biofilm in all isolates tested as mature biofilm. Our results agree with other reports. Montironi et al. (10) informed that EO and limonene were able to decrease biofilm production in *Streptococcus uberis* isolates. Similarly, Aiemsaard et al. (15) showed that limonene was able to inhibit biofilm formation as mature biofilm in *Staphylococcus aureus* isolates. Findings of the present study showed that higher inhibition percentages were obtained during biofilm formation, suggesting that both compounds were more effective at this stage.

## Conclusion

The results obtained in the present work contribute to the study of *Minthostachys verticillata* EO and limonene. Findings of the present report showed that EO affected the formation of biofilm and revealed the antibacterial capacity of EO and limonene suggesting its possible use as an alternative or complementary therapy in the control of bovine mastitis.

## Author contributions

ER and LC conceived and designed the experiments. MC performed all the experimental assays. MM contributed with experimental assays and statistical analysis. All the authors contributed to the manuscript writing and revision and they approved the final manuscript.

### Conflict of interest statement

The authors declare that the research was conducted in the absence of any commercial or financial relationships that could be construed as a potential conflict of interest.
